# *In-situ* and Real-Time Monitoring of the Interaction Between Lysins and *Staphylococcus aureus* Biofilm by Surface Plasmon Resonance

**DOI:** 10.3389/fmicb.2021.783472

**Published:** 2021-11-30

**Authors:** Wei Hong, Raphael Nyaruaba, Xiaohong Li, Huan Liu, Hang Yang, Hongping Wei

**Affiliations:** ^1^CAS Key Laboratory of Special Pathogens and Biosafety, Wuhan Institute of Virology, Center for Biosafety Mega-Science, Chinese Academy of Sciences, Wuhan, China; ^2^College of Life Sciences, University of Chinese Academy of Sciences, Beijing, China

**Keywords:** real-time, *S. aureus*, biofilms, lysins, CBD, CD, SPR, affinity

## Abstract

*Staphylococcus aureus* can produce a multilayered biofilm embedded in extracellular polymeric matrix. This biofilm is difficult to remove, insensitive to antibiotics, easy to develop drug-resistant strains and causes enormous problems to environments and health. Phage lysin which commonly consists of a catalytic domain (CD) and a cell-wall binding domain (CBD) is a powerful weapon against bacterial biofilm. However, the real-time interaction between lysin and *S. aureus* biofilm is still not fully understood. In this study, we monitored the interactions of three lysins (ClyF, ClyC, PlySs2) against culture-on-chip *S. aureus* biofilm, in real-time, based on surface plasmon resonance (SPR). A typical SPR response curve showed that the lysins bound to the biofilm rapidly and the biofilm destruction started at a longer time. By using 1:1 binding model analysis, affinity constants (*K*_D_) for ClyF, ClyC, and PlySs2 were found to be 3.18 ± 0.127 μM, 1.12 ± 0.026 μM, and 15.5 ± 0.514 μM, respectively. The fact that ClyF and PlySs2 shared the same CBD but showed different affinity to *S. aureus* biofilm suggested that, not only CBD, but also CD affects the binding activity of the entire lysin. The SPR platform can be applied to improve our understanding on the complex interactions between lysins and bacterial biofilm including association (adsorption) and disassociation (destruction).

## Introduction

Compared to microbiological laboratory conditions where *Staphylococcus aureus* often grows planktonically in nutrient-rich conditions, *S. aureus* found in the environment almost always forms sessile microbial communities called biofilms on both biotic and abiotic surfaces (Periasamy et al., 2012; Moormeier and Bayles, 2017). Increasing attention has been focused on *S. aureus* biofilms due to their relation to many human disease and recalcitrance to conventional antibiotics (Archer et al., 2011; Algburi et al., 2017). Combined with the spread of methicillin-resistant *Staphylococcus aureus* (MRSA), novel therapeutic strategies are in urgent need (Chen et al., 2013). Bacteriophage-encoded lytic enzymes, also known as lysins or endolysins, have been suggested as powerful weapons to combat *S. aureus* biofilms owing to their strong lytic activity, high specificity, and rare chance of resistance development (Fischetti, 2008; Szweda et al., 2012; Cha et al., 2019).

These specialized lysins usually have a modular structure containing two domains separated by a flexible linker region: one or more N-terminal catalytic domains (CD) for catalyzing peptidoglycan degradation and one C-terminal cell-wall binding domain (CBD) for recognizing and binding to specific cell wall ligands (Oliveira et al., 2013). Lysins can naturally exist in the environment (like PlySs2) or they can be engineered by shuffling the CBD or CD from different lysins to develop new chimeric lysins with enhanced properties (Schmelcher et al., 2011). For example, by fusing the CD of lysin Ply187 with the CBD of PlySs2, a novel chimeric lysin, ClyF, with increased thermostability, pH tolerance, and antibacterial activity against both planktonic and biofilm MRSA was formed (Yang et al., 2017). Similarly, ClyC, a novel chimeric lysin with increased bactericidal activity against *S. aureus* strains and its biofilms, including MRSA, in the presence of calcium ions was formed by fusing the CD of lysin Ply187 with the CBD of LysSA97 (Li et al., 2021). Despite the successful engineering of chimeric lysis, questions still remain such as how the lysins interact with the biofilms and if different CDs and CBDs could affect the ability of lysins to remove biofilms.

Currently, few methods can be used to measure lysin to biofilm interactions. Commonly used methods like crystal violet staining, bacterial viability counts, and electron microscopy are all based on endpoint quantification and cannot show the real-time process (Gutiérrez et al., 2014; Schuch et al., 2017). An impedance-based technology using the xCelligence real-time cell analyzer equipment is fast and reliable (Gutiérrez et al., 2016). However, this method only records biofilm clearance and also fails to show the process of lysins binding to biofilms which is the first and important step for lysin-biofilm interactions. This leaves a gap for exploring new analytical techniques for real-time monitoring of lysin-biofilm interactions.

Surface plasmon resonance (SPR) has emerged as a unique tool for label-free and real-time characterization and quantification of bio-molecular interactions (Schasfoort et al., 2018). Recently, SPR has also been applied to study the interaction between the secondary cell wall polysaccharides and phage lysins, as well as the interaction between phages and host bacteria (Tawil et al., 2012; Ganguly et al., 2013). However, to the best of our knowledge, no study has used SPR to investigate the dynamic process of lysin-biofilm interactions.

Hence, in this study, we aimed to show the real-time process of interactions between *S. aureus* biofilms formed on gold chip sensors and lysins with different CDs and CBDs based on SPR. The whole process of lysin-biofilm interaction, including association, disassociation and biofilm destruction was recorded in real-time for the first time. Based on the curves, the affinity between the biofilm and the lysins was calculated. These results demonstrated that SPR is a promising technique for monitoring the interactions between lysins and biofilm. The dynamic process revealed by SPR would help in understanding the antibiofilm property of lysins and developing better lysins for biofilm removal.

## Materials and Methods

### Protein Purification

The plasmid constructs and protein purification procedures were performed as in our previous studies (Huang et al., 2015; Yang et al., 2017; Li et al., 2021). Briefly, *E. coli* BL21(DE3) cells with their plasmids were grown in LB medium supplemented with 50 μg/mL kanamycin at 37°C 160 rpm to an OD600 of 0.4∼0.6. To allow for protein expression, the cells were further cultured for an additional 16 h at 16°C 120 rpm after induction with 0.2 mM isopropyl β-D-thiogalactoside. The cultured cells were then harvested by centrifugation at 6,000 × *g* for 10 min, resuspended with a suitable volume of 20 mM imidazole, lysed by a cell disrupter on ice, centrifuged at 10,000 × *g* for 30 min, and then filtered through a 0.22 μm syringe filter to remove cell debris. His-tagged proteins were purified by a HisTrap FF column (GE Healthcare, United States) by washing with 20, 40, and 60 mM imidazole, respectively, and finally eluting with 250 mM imidazole. Collected proteins were dialyzed against PBS buffer. The concentrations of the proteins were detected by a Pierce bicinchoninic acid (BCA) protein assay kit (Thermo Scientific, United States). The purity of each protein was determined by sodium dodecyl sulfate polyacrylamide gel electrophoresis (SDS-PAGE).

### Crystal Violet Staining Assays

The efficacy of lysins against *S. aureus* biofilm was determined by crystal violet (CV) staining assay. Briefly, *S. aureus* CCTCC AB91118 cells were cultured in 96-well polystyrene plates (Tissue culture treated, Nest, China) supplemented with TSB (Tryptic Soy Broth) containing 1% glucose (TSBG) for 24 h. Cultured cells were then washed three times with PBS to remove the planktonic cells and the resultant biofilms in each well treated with 200 μl 2.5 μM lysins at 25°C for 5, 10, 20, and 40 min, respectively. Afterward, wells were washed twice with PBS, air-dried, and then stained with 200 μl 0.1% CV (Merck, United States) for 5 min. Finally, after washing with PBS several times, each well was supplemented with 200 μl absolute ethanol and its OD595 measured by a microplate reader.

### Culturing Bacterial Biofilms on a Gold Chip

Au-sensor slides (gold chips) were purchased from BioNavis (BioNavis Ltd., Tampere, Finland). Each gold chip was cleaned with ammonia/hydrogen peroxide carefully and used for the experiment immediately after cleaning. Briefly, a solution of 1-part ammonia (30%) and 1-part hydrogen peroxide (30%) in 5-parts of water was boiled. A gold chip was then immersed in the boiling solution for about 10 min, rinsed with pure water, and then dried by gently blowing a stream of nitrogen over it. *S. aureus* (AB91118) and *Listeria monocytogenes* (ATCC 19115) cells were cultured with constant shaking at 160 rpm in 20 ml TSB at 37°C overnight, centrifuged, and then resuspend in fresh 20 ml TSBG. The gold chip was then placed in this bacterial suspension and cultured for another 24 h at 37°C without shaking. Post-culture, the chip was rinsed with PBS three times and blocked with 5% BSA for 1 h at 37°C. Finally, the gold chip was thoroughly rinsed with pure water and dried with nitrogen.

### Quantification of Culture-On-Chip Biofilms

Surface plasmon resonance was used to quantify the culture-on-chip biofilms and the experiments were recorded on a BioNavi-200 biosensor system (BioNavis Ltd., Tampere, Finland) at 25°C using PBS (pH 7.4) as the running buffer for all kinetic experiments. Experiments were conducted in the angular scan mode, with 58 – 78 degree ranges and monitored with a 670 nm laser source. To validate the stability of bacterial biofilms, PBS was flown over both channels at a flow rate of 30 μl/min for 80 min. For the evaluation of specific binding, serial dilutions (0.078, 0.156, 0.313, 0.625, 1.25, and 2.5 μM) of lysin ClyF were freshly prepared in PBS. A total volume of 150 μl was injected for each concentration at a flow rate of 30 μl/min. Dissociation was performed at 5 min intervals.

### Real-Time Monitoring of Biofilm-Lysin Interactions

Briefly, PBS was flown over the culture-on-chip *S. aureus* biofilm at a flow rate of 30 μl/min for several minutes to get a stable baseline and then replaced with 5 μM ClyF for another 30 min at the same flow rate. The results were observed and recorded in real-time using the TD driver software. To confirm the results, three additional tests (binding and biofilm destruction assays, and scanning electron microscopy (SEM)) were performed. For biofilm-ClyF binding assay, 200 μl of 5 μM ClyF was dropped onto the culture-on-chip *S. aureus* biofilm and 10 μl droplet was taken out every minute to measure ClyF concentration using the BCA method. For biofilm destruction detection, culture-on-chip *S. aureus* biofilm was treated with 5 μM ClyF for different minutes, washed quickly with PBS, and then put in 1 ml PBS. Released ATP was tested after treatment for 0, 1, 2, 3, 8, 16, 24, 32, and 40 min by an ATP detection kit (Scithera Ltd., Wuhan, China) following the manufacturer’s instructions. The structural changes of *S. aureus* biofilm on gold chips after ClyF injection was also observed by SEM. Here, 5 μM ClyF was flown over the culture-on-chip *S. aureus* biofilm at a flow rate of 30 μl/min for 0, 10, 20, and 25 min. The chips were then fixed with 2.5% glutaraldehyde and dehydrated by granted ethanol (from 30 to 100%). After treatment with critical point drying and sputter-coated with gold, the chips were finally analyzed by SEM (SU8010, Hitachi, Japan).

### Kinetic Evaluation

Three lysins were used to determine the binding kinetics between the lysins and the culture-on-chip *S. aureus* biofilms under two-fold serial dilutions, i.e., ClyF from 0.625 to 10 μM, ClyC from 0.156 to 2.5 μM, and PlySs2 from 2.5 to 40 μM. Briefly, 100 μl of each lysin solution was injected for 3.3 min, followed by PBS for a dissociation time of 7 min at a flow rate of 30 μl/min using single cycle kinetics. The binding kinetics (*K*_D_) for each lysin was then derived by fitting the experimental data at different concentrations to two interaction models (1:1 and 1:2) using the TD driver software. The two models used here have been previously described (Karlsson and Fält, 1997; Myszka et al., 1997) and we adapted them to test our binding interactions as follows:

(1) The 1:1 interaction model is simple and ordinary, describing one ligand (A) binding to one target (B) which leads to the formation of the conjugate (AB):


A+B⇔A⁢B

The interaction will fit the following equation:


(1) $$K_{\mathrm{D1}}=\frac{k_{\mathrm{d1}}}{k_{\mathrm{a1}}}=\frac{\left[A\right]\cdot \left[B\right]}{\left[A\,B\right]}$$

(2) 1:2 is an interaction model designed for one ligand (A) binding to two independent targets (B) and (C) which leads to the formation of two conjugates (AB) and (AC). It will always produce a better fit than 1:1 because it has greater flexibility.


A+B⇔A⁢B   A+C⇔A⁢C

This interaction model can be represented by two equilibrium equations:


(2) $$K_{\mathrm{D1}}=\frac{k_{\mathrm{d1}}}{k_{\mathrm{a1}}}=\frac{\left[A\right]\cdot \left[B\right]}{\left[A\,B\right]}\;K_{\mathrm{D2}}=\frac{k_{\mathrm{d2}}}{k_{\mathrm{a2}}}=\frac{\left[A\right]\cdot \left[C\right]}{\left[A\,C\right]}$$

In both Eqs.1, 2: [*A*][*B*][*C*][*AB*][*AC*] are concentrations of binding partners and conjugates; *K*_D_ (M) is the equilibrium dissociation constant (or affinity); *k*_D_ (s^–1^) is the kinetic dissociation constant (the rate of formation of conjugate); and *k*_*a*_ (M^–1^s^–1^) is the kinetic association constant (the rate of conjugate dissociation).

## Results

### *Staphylococcus aureus* Forms Stable Biofilms on the Sensor Chips

**FIGURE 1:**
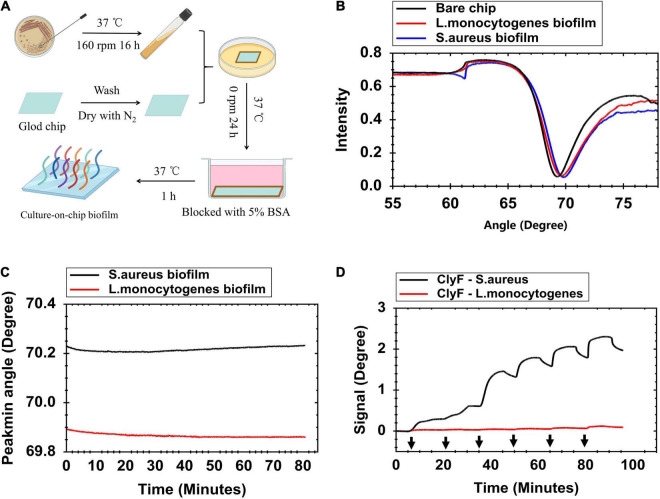
Formation and characterization of *S. aureus* biofilm on the gold sensor chip. **(A)** Schematic presentation of the culture-on-chip biofilm formation process. **(B)** Full angular spectrum of a clean gold surface (black line), *S. aureus* biofilm (blue line), and *L. monocytogenes* biofilm (red line) modified gold surface chip. **(C)** Contact angle change measurements when flowing PBS over the modified chip at 30 μl/min for 80 min to evaluate stability of biofilms. **(D)** SPR signal response for determining lysin adsorption on bacterial biofilm at increased concentrations of ClyF (black arrows from left to right mean 0.078, 0.156, 0.313, 0.625, 1.25, and 2.5 μM, respectively). “Signal” means change in Peakmin angle.

Gold has strong adsorption property. *S. aureus* tends to form biofilms on metal surfaces easily (Lee et al., 2015; Nan et al., 2015). These two reasons make it easy to culture bacterial biofilm on gold chips. Figure 1A describes the steps for forming the culture-on-chip biofilms. Three important steps should be considered during preparation: (1) The chips must be cleaned with ammonia/hydrogen peroxide firstly; (2) High concentrations of *S. aureus* need to be used to form stable biofilms; and (3) Blocking with BSA is a necessary step to saturate non-specific protein absorption sites on the chip. In order to quantify the biofilm, full angular scan reflectivity spectra were measured first by SPR and it was found that the biofilm binding shifts the resonance to a higher angle (∼0.5 degree) compared with the bare chip (Figure 1B). This shift in resonance angle was maintained even after three repeats as shown in Supplementary Figure 1A. The stability of the formed bacterial biofilms was tested by running buffer (PBS) flown over the chip surface at a flow rate of 30 μl/min for 80 min. Under this test pressure, the culture-on-chip biofilm resulted in a baseline with acceptable stability (less than 0.05-degree decline) (Figure 1C). To evaluate the biofilm functionality, increased concentrations of ClyF (0.078 – 2.5 μM) were injected. Dose-dependent binding response curves were obtained for ClyF-*S. aureus* biofilm, but not for ClyF-*L. monocytogenes* biofilm (Figure 1D). Due to competitive inhibition, the signal difference decreased after each injection of ClyF from low concentrations to high concentrations (Figure 1D). From this, repeated testing was done on *S. aureus* biofilms only and the results were highly reproducible as shown in Supplementary Figure 1B.

### Real-Time Analysis of Culture-On-Chip Biofilm Interactions With Lysin

**FIGURE 2:**
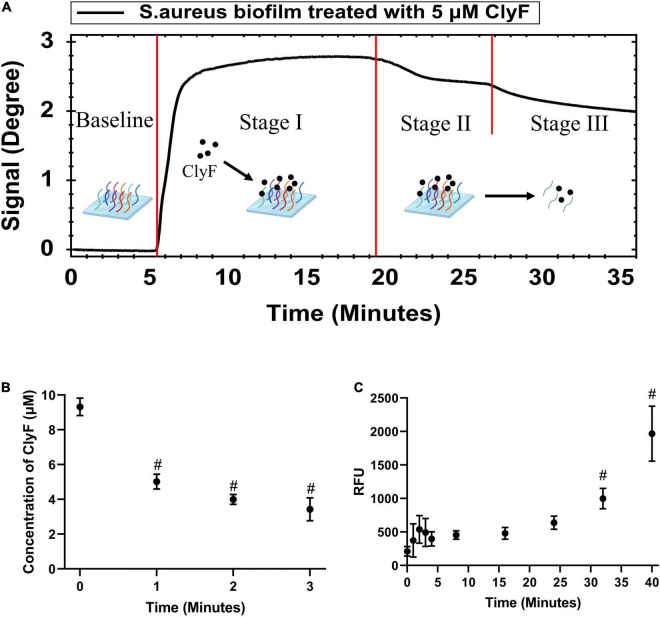
Real-time monitoring and confirmation of cultured-on-chip *S. aureus* biofilm interactions with lysin ClyF. **(A)** Representative SPR sensorgram illustrating changes in Peakmin Angle after lysin injection. **(B)** Monitoring the absorption process by detection of free ClyF concentration. **(C)** Monitoring the biofilm destruction process based on released ATP by ATP detection. # denotes *p* < 0.05.

SPR was used to monitor the culture-on-chip *S. aureus* biofilm interactions with the lysin ClyF in real-time. As shown in Figure 2A and Supplementary Figure 2A, after 5 μM-ClyF was flown continuously over the culture-on-chip *S. aureus* biofilm, one adsorption stage (Stage I) and two dissociation stages (Stage II/III) were obtained. Following the baseline, in Stage I, a sharp rise in the curve was observed within the first 3 min that leveled off for the next 13 min due to ClyF adsorption onto the *S. aureus* biofilm. However, for Stages II/III, we could not clearly distinguish between the two dissociation stages because both exhibited a recession curve. The real-time process of lysins acting on the culture-on-chip biofilm was further confirmed by three independent experiments. In the first one, 200 μl of 5 μM ClyF was dropped onto the culture-on-chip biofilm and the free ClyF concentrations were detected using BCA over time to determine adsorption. As seen in Figure 2B, a sharp decline in the concentration was observed within the first 3 min, indicating that ClyF was quickly adsorbed by the culture-on-chip biofilm. This confirmed Stage I of Figure 2A. In the second experiment, ATP released as a result of biofilm interaction with ClyF was measured using a luminometer to determine biofilm destruction. As shown in Figure 2C, biofilm cleavage began at about 32 min after 5 μM ClyF treatment. This experiment, however, did not completely simulate the real-time SPR experiment mainly because in the SPR experiment, ClyF was flown through bacterial biofilm at a constant flow rate of 30 μl/min, while in this experiment the process was static. Hence, to clearly determine and distinguish stages II and III, SEM was performed at the different stages as shown in Supplementary Figure 2B. Since SEM cannot be used to observe the lysin, we observed the structural changes of the biofilm at different times. After 30 min, we could observe biofilm destruction which may be the possible reason for the declining curve. This time corresponds to stage III of the sensogram curve.

### Binding Model of Lysin to Culture-On-Chip Biofilm

**FIGURE 3:**
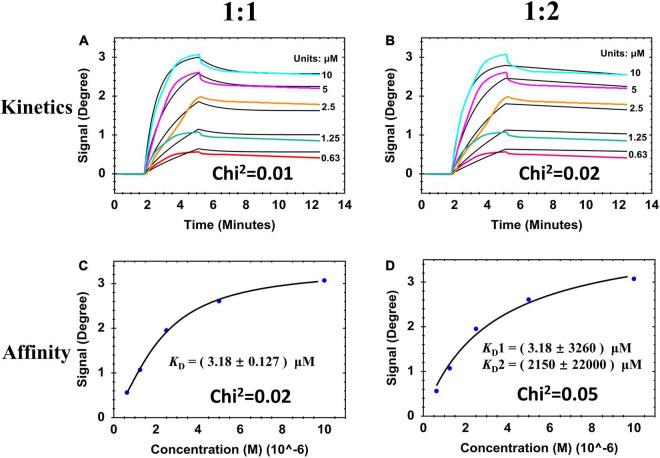
SPR sensorgrams and resulting affinity fit curves for ClyF binding to cultured-on-chip *S. aureus* biofilm analyzed by two binding models responding to 1:1 and 1:2. **(A,B)** Sensorgrams of the binding curves (colored lines) and the fitted data (black lines). **(C,D)** Corresponding plots (blue dots) and fitted data (black lines) of steady-state binding from the end of the association phases against two-fold serially diluted concentrations.

When analyzing the binding activity between lysins and biofilm, injection time should be controlled or else biofilm destruction will occur. Following the SPR results from Figure 2, injection of 100 μl lysins at a flow rate of 30 μl/min was chosen as the test condition. Under this condition, the binding interaction was described using two typical curves: (1) Sensorgram curve (Figures 3A,B) that was time-dependent and showed how fast binding happens; and (2) Saturation curve (Figures 3C,D) that was time-independent and showed how strong the binding complex is. We further attempted to fit these response curves using the two binding models 1:1 and 1:2 (Methods section Kinetic Evaluation). By observing the deviation between the fit curves (black lines in Figure 3), measured data (colored lines in Figures 3A,B and blue dots in Figures 3C,D), Chi-square and *K*_D_ values (inset values in each Figure), a relatively good model (1:1) was chosen for further experiments. The 1:1 binding model had better fit curves lower Chi-square values, and its *K*_D_ had the lowest standard deviation compared to the 1:2 binding model. This results were consistent with previous reports (Gutiérrez et al., 2016; Koponen et al., 2020).

### Binding Affinity of Lysin to Biofilm

**FIGURE 4:**
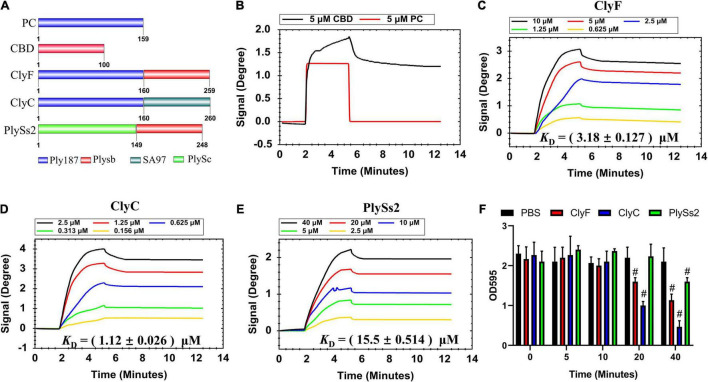
Determination of lysin binding affinity to *S. aureus* biofilm. **(A)** Schematic representation of lysins and their constituents. Sensorgrams depicting **(B)** CBD and PC; **(C)** ClyF; **(D)** ClyC; and **(E)** PlySs2 dose-dependent binding to culture-on-chip *S. aureus* biofilm. **(F)** Biofilm removal efficacy of 2.5 μM lysin (ClyC, ClyF, PlySs2) and PBS over time. # denotes *p* < 0.05.

To study the influence of CBDs and CDs on lysin binding affinity, we used three previously reported lysins (ClyF, ClyC, and PlySs2) (Huang et al., 2015; Yang et al., 2017; Li et al., 2021). From the schematic representation of these lysins (Figure 4A), both lysins ClyF and ClyC shared the same CD (i.e., PC, 159 amino acids) while lysins ClyF and PlySs2 shared the same CBD (i.e., Plysb, 100 amino acids). We also purified the two constitutive domains of ClyF, i.e., PC and CBD for further studies. As shown in Figure 4B, both 5 μM CBD and 5 μM PC could cause SPR signal change in the loading stage (∼2 min). However, in the PBS elution stage (∼5.3 min), the PC signal rapidly dropped to the baseline. Although CBD signal dropped from 1.7 degree to 1.3 degree in the same stage, it remained at 1.3 degree for the next 6 min. This result suggested that the CBD could bind to *S. aureus* biofilm, whereas PC could not. Three binding data sets for the lysins ClyF, ClyC, and PlySs2 are shown in Figures 4C–E, respectively. In each data set, lysins with two-fold diluted concentrations were used and described in different colors. In order to make the calculation more accurate, different concentrations (0.625 – 10 μM for ClyF; 0.156 – 2.5 μM for ClyC; and 2.5 – 40 μM for PlySs2) for different lysins were used to cover a full range of the binding curves. After analyzing affinity constants by the 1:1 binding model, the *K*_D_ for ClyF, ClyC, and PlySs2 were 3.18 ± 0.127 μM, 1.12 ± 0.026 μM, and 15.5 ± 0.514 μM, respectively. Hence, the affinity strength decreased in the order: ClyC > ClyF > PlySs2. Combined with the structural information of these lysins (Figure 4A), it seemed that both constitutive domains of lysins affected its binding affinity to bacteria. Furthermore, we compared the biofilm removal efficacy of the three lysins using CV staining. In this experiment, the OD595 represented the biofilm mass. As shown in Figure 4F, about 50% of the *S. aureus* biofilms were disrupted after exposure to 2.5 μM ClyC for 20 min. While at the same condition, the removal efficacy was 19% and 1% for ClyF and PlySs2, respectively. These results were consistent with the affinity strength results as in both assays the efficacy of these lysins decreased in the order: ClyC > ClyF > PlySs2.

## Discussion

A major shortcoming in the study of anti-biofilm agents is lack of real-time monitoring techniques. Common approaches, like crystal violet staining and microscopy, are time-consuming, end-point, and label-dependent (Tremblay et al., 2015). Other real-time methods like impedance-based technology can only monitor changes in biofilm mass after lysin treatment (Gutiérrez et al., 2017) while ignoring other important steps like lysin-biofilm interactions. SPR experiments can record the whole process of lysin-biofilm interaction, including association and disassociation, and calculate the kinetic parameters in real-time. Additionally, culture-on-chip *S. aureus* biofilm techniques can effectively fill the gap of SPR application in studying bacteria-protein interaction, because protein-protein (Lê et al., 2010) and mammalian cells-protein (Koponen et al., 2020) interaction are already widely reported.

In this study, we show the real-time process of lysin-biofilm interactions using SPR. From our results, three typical SPR sensograms are of interest. In the first one (Figure 1D), its shown that biofilms can stably interact with increased lysin concentrations. This results in a sensorgram that suggests the existence of competitive inhibition, i.e., continuous loading of ClyF from 0.078 to 2.5 μM does not mean that the higher the concentration loaded, the higher the SPR signal observed. Instead, the SPR signal increases significantly at lower concentration ranges. This means that SPR can also be applied to study the competitive and cooperative relationship between two analytes as in previous studies (Yao et al., 2021). The second sensogram (Figure 2A) which is our main finding, shows the real-time process of lysin-biofilm interaction. In this sensogram, lysin adsorption which results in a sharp rise in the curve is the main process observed. The lysin-biofilm removal process is, however, indistinguishable but evident in the curve (i.e., a weak signal resulting in a 6-degree decline from 20 to 35 min). This decline may be caused by lysed bacterial clusters that are still covered on the SPR chip as previously reported (Obořilová et al., 2021) and observed by SEM in Supplementary Figure 2. In the SEM result, further lysis results in destruction of the biofilm which may characterize stage III of the sensogram. Despite this, the lysed biofilm is still on the surface of the chip hence the curve will not drop completely. The last sensograms (Figure 4) show that lysins can be used to calculate some kinetic binding parameters. The best concentration range is around 0.1 -- 10 times the *K*_D_ because high concentration will tend to make the curves to bind together, and low concentration will give a low response curve^1^. *K*_D_s between *S. aureus* biofilm and endolysins ranged from 1.12 to 15.5 μM, similar to Ganguly’s et al. study which reported that the endolysins PlyL and PlyG bind to the secondary cell wall polysaccharides (SCWPs) from *Bacillus anthracis* with *K*_D_s ranging from 0.81 to 7.51 μM (Ganguly et al., 2013). In addition, Yang et al. (2017) reported that 3.3 μM ClyF significantly decreased *S. aureus* biofilm mass by 25.2%. Interestingly, this treatment concentration is quite close to its *K*_D_ (3.18 ± 0.127 μM) as shown in this study.

Classical lysins require CBD that directs CD to peptidoglycan layer. Actually, the functions of these two domains are not independent of each other. At present, there are several consensus on the relationship between CBD and CD: (1) CBD largely determines the lytic spectrum and CD is involved in peptidoglycan lysis (Broendum et al., 2018; Binte Muhammad Jai et al., 2020); (2) Engineering positively charged CD-only lysin could eliminate CBD dependence and possess a broader lytic spectrum (Low et al., 2011); and (3) Linker editing between CBD and CD can have minor effects on the folding of these two domains and improve their lytic activity (Yang et al., 2020). Our data suggests that both CBD and CD can affect the binding activity of lysins by comparing their *K*_D_s (Figure 4). These *K*_D_s may be used as another important index to evaluate the bactericidal activity of lysins and help create more active lysins with better combinations.

Despite the successful real-time monitoring of *S. aureus* biofilm and lysin interactions, it is also important to note that biofilms cultured on the gold chips may be different from those in nature. Additionally, in this study, we only chose one *S. aureus* strain. More MRSA strains and clinical isolates should be studied to further reveal the true potential of SPR in monitoring lysin-biofilm interactions.

In conclusion, for the first time, a real-time biosensing platform based on SPR technology was used to monitor the interactions between bacterial biofilm and lysins. The whole process revealed that lysin ClyF could bind to *S. aureus* biofilm rapidly, but the biofilm destruction process needs a longer time to start. The SPR platform can be applied to reveal the complex interactions between lysins and bacterial biofilms including association, disassociation and biofilm destruction, and also calculate their kinetic parameters.

## Data Availability Statement

The original contributions presented in the study are included in the article/Supplementary Material, further inquiries can be directed to the corresponding authors.

## Author Contributions

WH, HY, and HW designed the study. WH performed the experiments. HW, RN, XL, and HL performed data analysis. HY and HW contributed with reagents and funds for research. HW and RN wrote the draft manuscript. HW, RN, and HY revised the manuscript. All authors contributed to the article and approved the submitted version.

## Conflict of Interest

The authors declare that the research was conducted in the absence of any commercial or financial relationships that could be construed as a potential conflict of interest.

## Publisher’s Note

All claims expressed in this article are solely those of the authors and do not necessarily represent those of their affiliated organizations, or those of the publisher, the editors and the reviewers. Any product that may be evaluated in this article, or claim that may be made by its manufacturer, is not guaranteed or endorsed by the publisher.

## Footnotes

1
https://www.sprpages.nl/sensorgram-tutorial/a-curve
