# Supplementary material for: In-situ and Real-Time Monitoring of the Interaction Between Lysins and Staphylococcus aureus Biofilm by Surface Plasmon Resonance
Source: Front Microbiol. 2021 Nov 30;12:783472. doi: 10.3389/fmicb.2021.783472 (PMC8670000; doi:10.3389/fmicb.2021.783472)
Supplement: Supplementary file 1 [file Table_1.DOCX]

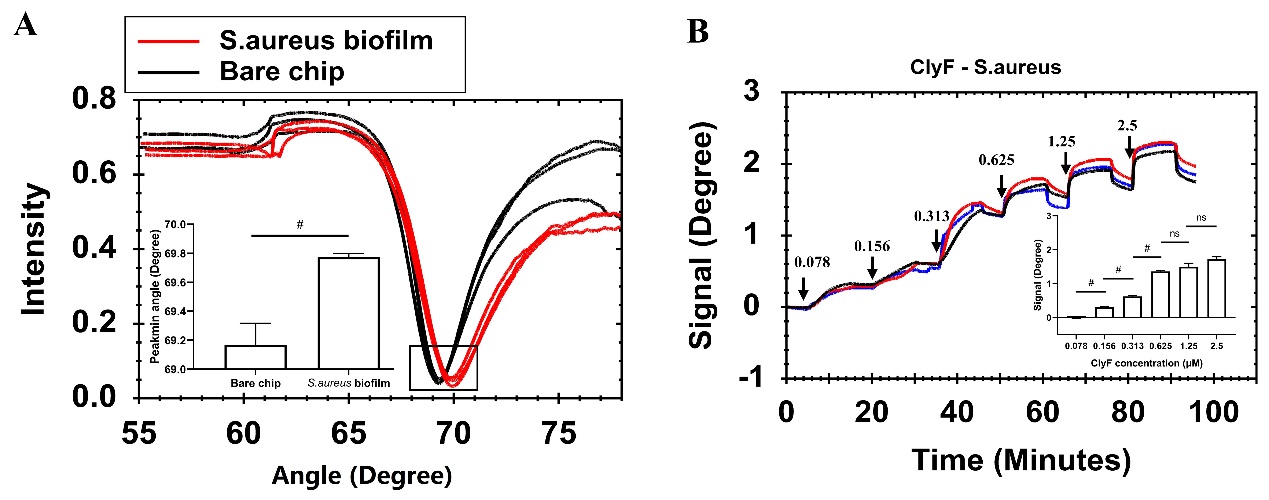


**Figure S1.** Characterization of *S.aureus* biofilm on the gold sensor chip in triplicate. (A) Full angular spectrum of a clean gold surface (black lines) and *S.aurues* biofilm modified gold surface chip (red lines). The histograms of the inset chart show the change in peakmin angle between the two surfaces. (B) SPR signal response of culture-on-chip biofilm treated with increased concentrations of ClyF. Black arrows and upper values represent the different concentrations in μM added. The histograms of the inset chart show signals at injected concentration changes. Data are shown with mean ± SD. Error bars represent the standard deviations for triplicates. # denotes p < 0.05. ns denotes not significant.


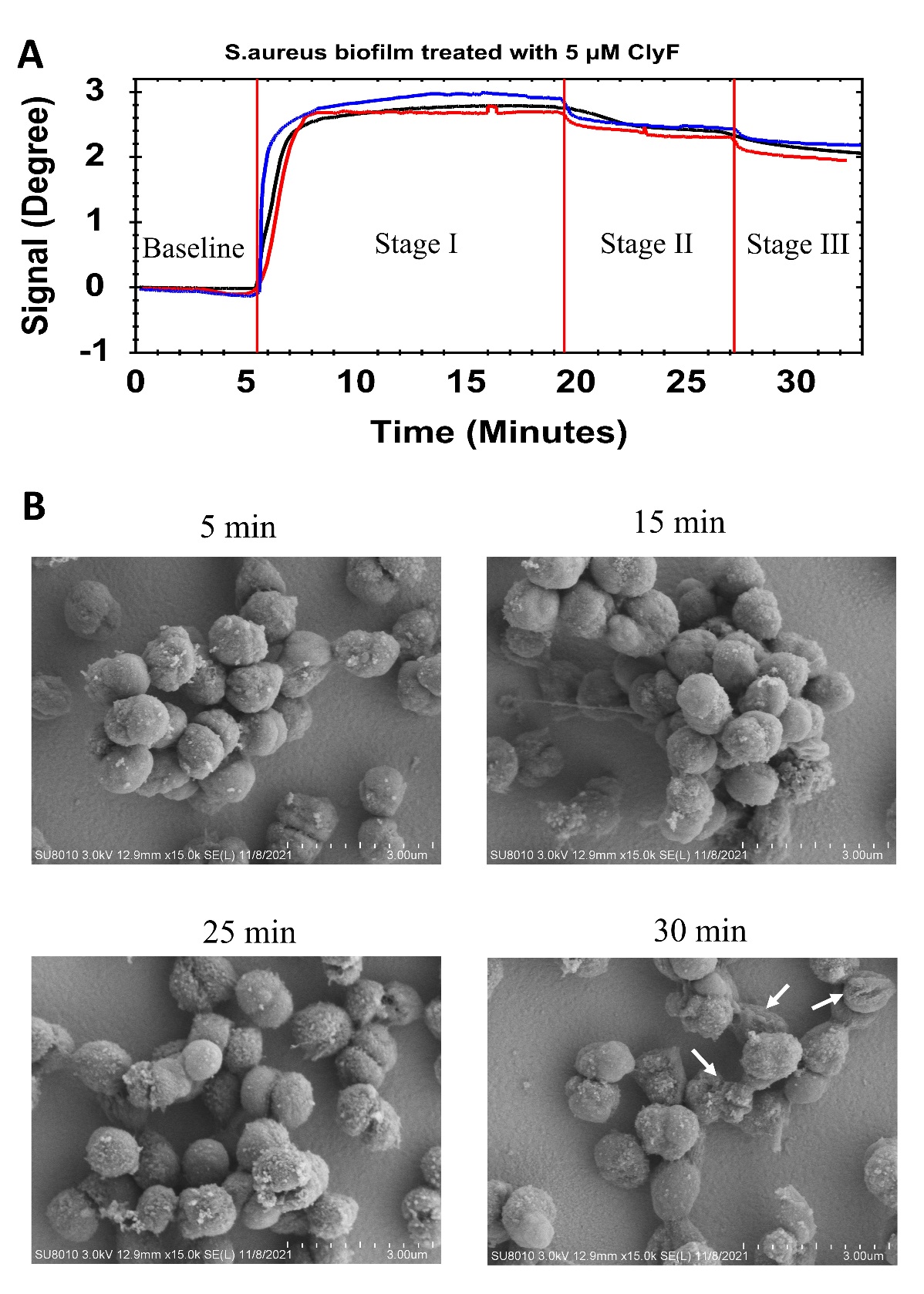


Figure S2. Real-time monitoring and confirmation of culture-on-chip *S.aureus* biofilm interaction with 5 μM ClyF by SPR and SEM. (A) Triplicate SPR sensorgram curves illustrating changes in signal after ClyF was injected. (B) SEM analysis of culture-on-chip biofilm with increased treatment time. The photographs were taken at various time points to establish the lysin-biofilm interactions on the gold surface chips. The white arrows show *S. aureus* biofilm destruction.
